# Role of IGF1R in Breast Cancer Subtypes, Stemness, and Lineage Differentiation

**DOI:** 10.3389/fendo.2015.00059

**Published:** 2015-04-24

**Authors:** Susan M. Farabaugh, David N. Boone, Adrian V. Lee

**Affiliations:** ^1^Department of Pharmacology and Chemical Biology, University of Pittsburgh, Pittsburgh, PA, USA; ^2^Women’s Cancer Research Center, Magee-Womens Research Institute, University of Pittsburgh Cancer Institute, University of Pittsburgh, Pittsburgh, PA, USA; ^3^Department of Human Genetics, University of Pittsburgh, Pittsburgh, PA, USA

**Keywords:** IGF1R, breast cancer subtypes, lineages, luminal, ERBB2^+^, ER^+^, triple negative

## Abstract

Insulin-like growth factor (IGF) signaling is fundamental for growth and survival. A large body of evidence (laboratory, epidemiological, and clinical) implicates the exploitation of this pathway in cancer. Up to 50% of breast tumors express the activated form of the type 1 insulin-like growth factor receptor (IGF1R). Breast cancers are categorized into subtypes based upon hormone and ERRB2 receptor expression and/or gene expression profiling. Even though IGF1R influences tumorigenic phenotypes and drug resistance across all breast cancer subtypes, it has specific expression and function in each. In some subtypes, IGF1R levels correlate with a favorable prognosis, while in others it is associated with recurrence and poor prognosis, suggesting different actions based upon cellular and molecular contexts. In this review, we examine IGF1R expression and function as it relates to breast cancer subtype and therapy-acquired resistance. Additionally, we discuss the role of IGF1R in stem cell maintenance and lineage differentiation and how these cell fate influences may alter the differentiation potential and cellular composition of breast tumors.

## IGF Signaling

Insulin-like growth factor 1 and 2 (IGF1 and IGF2) are peptides that act as circulating endocrine hormones critical for normal body growth. Pituitary-derived growth hormone (GH) stimulates the liver to express IGF1, which is secreted and affects growth of multiple cell types (1, 2). Other tissues also produce IGF ligands that act in a paracrine or autocrine manner (3). IGF ligands are bound by insulin-like growth factor binding proteins (IGFBP1-6) (4). While IGFBP binding increases ligand stability, IGFBP binding also decreases ligand bioavailability and competes with ligand-receptor binding.

Insulin-like growth factor 1 or IGF2 stimulates downstream signaling events primarily by binding and activating the type 1 insulin-like growth factor receptor (IGF1R). Insulin-like growth factor type 2 receptor (IGF2R), which is homologous to the mannose-6-phosphate receptor, does not seem to have a signaling function and may act as a sink to modulate IGF2 ligand bioavailability. IGF1R has high similarity to the insulin receptor (InsR) (5, 6). Indeed, although the affinities are much lower, IGF1 can bind and activate the InsR while, in turn, insulin can bind and activate IGF1R (7). IGF2 binds both receptors with similar affinities. In addition, a fetal form of InsR that has alternate splicing, termed as insulin receptor isoform A (InsR-A), has a high affinity for IGF2. IGF1R and InsR exist primarily as heterotetramers. However, hybrid IGF1R/InsR receptors, consisting of subunits from both receptors, can form and bind all three ligands (8–13). Therefore, there is much crosstalk and overlap in receptor signaling downstream of IGFs and insulin.

Binding of IGF1 or IGF2 to IGF1R results in auto-phosphorylation of the IGF1R kinase domain and activation of intracellular signaling cascades. Insulin receptor substrates 1 and 2 (IRS1 and IRS2), the main signaling adaptors for both IGF1R and InsR, are recruited to the receptor and act as docking sites (14). IRS1 and IRS2 link the activated receptors to numerous intracellular adaptor proteins and downstream signaling cascades such as PI3K/AKT and RAS/MAPK/ERK1 (15).

Type 1 insulin-like growth factor receptor signaling cascades regulate cell growth, survival, and motility. The IGF1R pathway mediates strong anti-apoptotic signals through three known pathways. The PI3K/AKT and RAS/MAPK pathways both facilitate IGF1R-induced resistance to apoptosis (16). Additionally, IGF1R interacts with 14.3.3 proteins to induce the mitochondrial translocation of Raf (17). All three of these pathways converge to phosphorylate BAD and block apoptosis. Additionally, IGF1R mediates the cell cycle through MAPK/ERK activation. ERK induces proliferation through phosphorylation of transcription factors such as c-Fos and Ets-like transcription factor 1 (Elk-1) (18). IGF1R signaling also activates c-Myc, JNK, and c-Jun (19). Cell cycle progression is promoted by IGF1R-mediated increases in ribosome activity (20) and expression of cyclins A, B, and D1 (21, 22). These robust anti-apoptotic properties of IGF1R and cell cycle regulation both play critical roles in promoting IGF1R-mediated tumorigenesis.

## IGF1 Signaling and Mammary Gland Function

Insulin-like growth factor 1 is critical for mammary development. In the mammary gland, GH stimulates IGF1 production from the stroma (23), and this is enhanced by estradiol (24, 25). IGF-I acts in a paracrine manner to stimulate terminal end bud (TEB) growth and form the ductal structures that extend through the mammary fat pad. Mammary gland development is decreased in IGF1 (−/−) null females (26). Interestingly, IGF1 produced within the mammary gland is more potent at stimulating mammary growth than circulating IGF1 derived from the liver (27). The IGF1 deficiency in IGF1 (−/−) null mice can be rescued by continuous 5-day injections of the des-IGF1 variant. Longer treatments with IGF1 plus estradiol enhance TEB formation and ductal morphogenesis. Interestingly, treatment with GH and estradiol does not restore development in IGF1-null mice, indicating the necessity of IGF1 for the full function and stimulation by these hormones (26).

Similar to the critical role for IGF1 in mammary gland development, IGF1R is also required for mammary gland morphogenesis. Reconstitution assays using IGF1R-deficient embryonic mammary buds demonstrate decreased growth potential and cell proliferation during morphogenesis (28). The reduction in mammary gland morphogenesis observed upon loss of IGF1/IGF1R signaling is a result of decreased cell cycle progression and increased apoptosis (29–31).

Although critical for mammary gland development, IGF1 needs to function in cooperation with growth factors such as epidermal growth factor (EGF) and transforming growth factor beta (TGF-B) as well as the IGF1 receptors and binding proteins for efficient signaling and full mammary function (32).

## The IGF Pathway in Cancer

As IGF1 plays such a critical role in cell growth, survival, and migration, it is not surprising that alterations in the IGF1 signaling pathway are linked to the development and progression of multiple cancers including breast, lung, osteosarcoma, gynecological, prostate, and gastrointestinal cancers. Many different alterations in the IGF system promote tumorigenesis: increased IGF1 and IGF2 expression (33–36), decreased levels of circulating IGFBPs (which increase ligand bioavailability) (37–39), and changes in receptor expression (40, 41).

**Figure 1:**
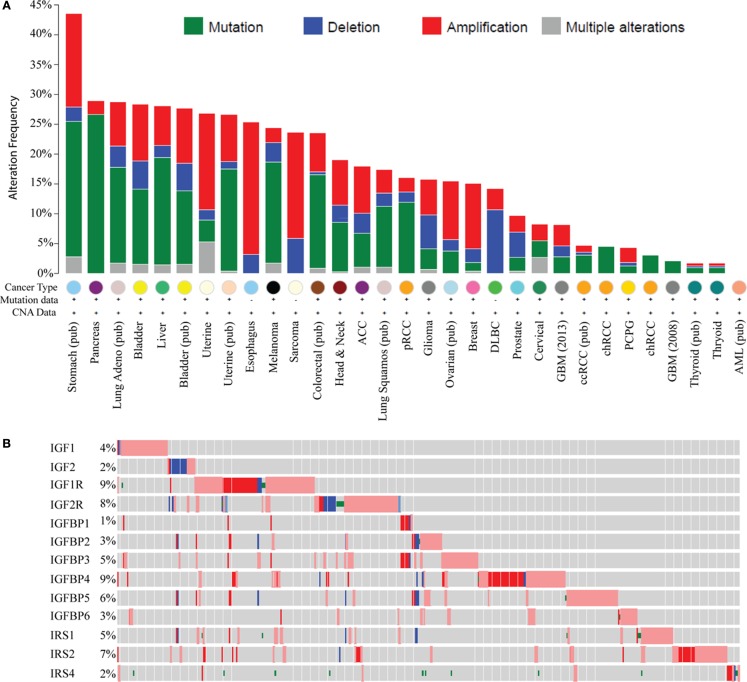
**Genomic and transcriptomic alterations in the IGF pathway in cancer**. Data are from The Cancer Genome Atlas at cBio (http://www.cbioportal.org/) in February 2015. For several cancers, the data are published (pub) while for others it is provisional and in progress. **(A)** Histogram showing genomic changes (somatic base pair mutation and copy number alteration) in 13 members of the IGF pathway [notated on the *y*-axis in **(B)**]. Each bar represents the percentage of tumors, which show an alteration in these genes and each cancer type is indicated on the *X*-axis. **(B)** Genomic and transcriptomic changes (mRNA levels) specifically in breast cancer for 13 genes indicated on the *y*-axis. Each row represents a different gene. Each gray box represents an individual tumor. The percentage of tumors showing an alteration for each gene is shown on the *Y* -axis next to the gene. A red box indicates the gene is amplified and blue is deleted. A box with a red outline represents RNA overexpression compared to normal breast and blue is underexpression. A green dot represents a somatic base pair mutation. Tumors without an alteration in any of the IGF family members have been removed for visualization.

Recent large genomic analyses now allow a comprehensive examination of genomic and molecular changes in the IGF pathway in cancer. Analysis of data from The Cancer Genome Atlas (TCGA) using the cBIO portal across most cancer types shows genomic alterations in IGF ligands (IGF1, 2), receptors (IGF1R, IGF2R), binding proteins (IGFBP1–6), and IRSs (IRS1, 2, 4); this representing 18 genes (Figure 1A). The greatest change is in stomach cancer where 43% of tumors show a molecular alteration (amplification, deletion, or base pair mutation) in one or more of the 18 genes of the IGF family. Interestingly, different tumor types show distinct molecular changes. For example, sarcomas exhibit copy number changes such as DNA amplification and deletion, whereas pancreas, melanoma, and lung cancers are dominated by base pair mutations. Some tumor types display a very low to absent mutation rate such as thyroid, acute myelogenous leukemia, and glioblastoma.

In breast cancer, 15% of TCGA-documented breast cancers (42) contain genomic alterations in the IGF pathway. These alterations consist mainly of amplification and are generally rare with only IGF1R and IRS2 showing amplification in >5% of cases. When considering mRNA levels (using the TCGA provisional data on 962 breast cancers which includes RNA-seq), 45.3% of breast cancers (*n* = 436/962) show a molecular alteration in at least one IGF family member. IGFIR is amplified, overexpressed, or somatically mutated in 9% of tumors. Other notable changes are amplification or overexpression of IGFBP4 (9%), IGFBP5 (6%), and IRS2 (7%) (Figure 1B).

While somatic mutation of IGF family members has only recently been comprehensively described, a large literature has reported on germline polymorphisms. In particular, germline polymorphisms in IGF1, IGF1R, and IGFBP3 are associated with increased risk of breast (43–45), prostate (46–48), lung (49), and pancreatic (50) cancers.

While many studies have examined alterations in IGF pathway components, few measured pathway activity. For example, tumor tissue microarrays show that 87% of primary breast tumors express IGF1R (51); however, the active phosphorylated form of IGF1R/InsR, as measured by immunohistochemistry (IHC), is only present in roughly 50% of breast cancers where it correlates with poor survival (52). We examined IGF pathway activity by combining IGF-regulated mRNA levels into an “IGF gene signature.” Breast tumors expressing the IGF1 gene signature significantly correlate with numerous poor prognostic factors and expression of this signature is one of the strongest indicators of poor disease outcome (53).

## IGF1R Across Breast Cancer Subtypes

Breast tumors display tremendous heterogeneity among different patients due at least in part to varying molecular alterations and divergent cells of origin. In recent years, gene expression profiling has helped to define breast cancer subtypes. Molecular profiling divides breast tumors into six major subtypes, which are related to the known major drivers (and targetable biomarkers): estrogen receptor α (ERα), progesterone receptor (PR), and erbb2 receptor tyrosine kinase 2 (ERBB2/HER2). The current molecular subtypes include luminal A, luminal B, ERBB2-like, triple negative/basal-like, claudin-low, and normal-like (54, 55). Each subtype can also be further classified into more defined subgroups.

**Figure 2:**
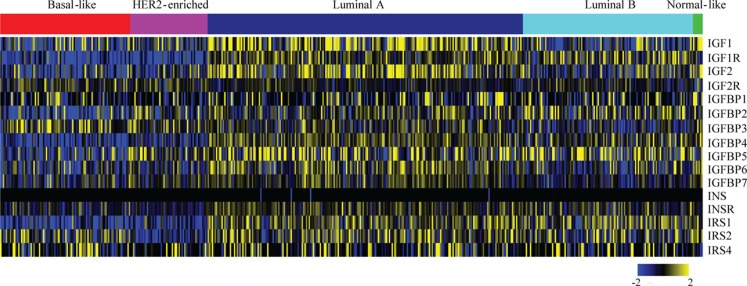
**Expression levels of IGF pathway components across breast cancer subtypes**. Level 3, IlluminaHiSeq_RNASeqV2 normalized gene expression values for all breast cancer tumors were downloaded from The Cancer Genome Atlas. The expressions of the indicated genes from tumors (columns) with calculated PAM50 scores (42) were extracted, log2 transformed, median centered for each gene (rows), and a heatmap was generated using MeV. The PAM50 subtype clusters are shown above with the indicated colors.

The expression and role of IGF1R in various breast cancer subtypes, and in particular its role in causing resistance to targeted therapies, has been extensively studied. By identifying in which tumor types the IGF1R pathway actively drives tumor initiation and progression, we can better define the subtype(s) that may benefit from anti-IGF1R therapies. For an overview of IGF pathway expression across the breast cancer molecular subtypes, we analyzed TCGA data (42) (Figure 2). Most IGF pathway members, including IGF1R itself, tend to be more highly expressed in luminal A and luminal B tumors and comparatively underexpressed in basal and ERBB2^+^ tumor types (Figure 2). This expression only denotes mRNA levels and does not demonstrate pathway activation.

Below, we describe the main breast cancer molecular subtypes and the role IGF1R is believed to play in each.

### Luminal

The majority of breast cancers fall into the luminal classification. Luminal subtypes tend to be hormone receptor positive (ER^+^, PR^+^) and comprise ~50% (luminal A) and ~10–20% (luminal B) of all breast cancer cases (56). Luminal A tumors have the best overall prognosis. Luminal B tumors are similar to luminal A tumors, but are more aggressive (higher grade) and are typically diagnosed at a younger age with higher reoccurrence rates. Hormone therapies (e.g., tamoxifen and aromatase inhibitors) have greatly increased the overall prognosis of the luminal subtype.

Estrogen receptor α is a major regulator of IGF signaling, due in part to transcriptional activation of IGF1R and many other IGF signaling components such as IRS1 (57–59). Consistent with this, the hormonally driven luminal subtypes tend to have higher levels of IGF1R and IRS expression as opposed to tumors that are less hormonally driven (triple negative and ERBB2^+^) (60, 61). IGF1R is expressed in 52 and 84% of Luminal A and 57.5 and 76% of Luminal B tumors, respectively (62, 63). IGF1R expression does not affect breast cancer specific survival in luminal A tumors. Interestingly, luminal B tumors with higher total IGF1R levels have significantly better prognosis than those with low levels of IGF1R (62). Law et al. demonstrated that roughly 50% of all luminal tumors show phosphorylated, and presumably active, IGF1R (52). As IGF1R is upregulated by ERα, the better prognosis of IGF1R-expressing luminal tumors may be associated with the use of ER-targeted therapies.

Estrogen receptor α and the IGF pathway show dynamic and intricate crosstalk, resulting in bidirectional regulation of expression and activity (64). ERα transcriptionally upregulates IGF1R expression. IGF1R transcriptionally upregulates ERα in an mTOR/S6K1-dependent manner and increases ERα phosphorylation to stimulate transcriptional activity (65, 66). Importantly, during endocrine resistance, converging growth factor signaling on the PI3K/AKT and MAPK/ERK pathways bypass the need for ERα activity (67–69). Surprisingly, however, use of anti-IGF1R therapy in the setting of endocrine resistance does not improve prognosis (70).

Consistent with its ERα-dependent regulation, IGF1R levels are reduced in many tamoxifen- and aromatase inhibitor-resistant cell and mouse tumor models (71–73) as well as patient tumors (74). However, the remaining IGF1R is strongly phosphorylated with hyperactive IGF1R/InsR/PI3K/AKT/mTOR signaling beyond pre-resistance levels, suggesting that the cells/tumors acquire resistance through an IGF1R-directed mechanism even in cases of reduced IGF1R levels (69, 73). Furthermore, the Yee lab has shown that while IGF1R is reduced in tamoxifen resistant breast cancer cells, InsR is still expressed and able to signal via insulin to promote growth (75).

Recently, the G protein estrogen receptor 1 (CPER/GPR30) has been identified as a potential mediator of rapid estrogens response. Increased GPER expression is associated with increased risk of metastasis and poor survival (76). Both IGF1 and insulin upregulate GPER expression through the c-Fos/AP1 pathway. IGF1 and insulin transactivate GPER to promote migration and proliferation (77, 78). ERα is required for IGF1-induced transactivation of GPER (77). Interestingly, GPER expression increases in tumors treated with tamoxifen (79) and correlates with a poorer prognosis specifically in tamoxifen treated patients (79–82). These results suggest that GPER may be a potential pathway for IGF1- and insulin-induced tamoxifen resistance.

Given basic signaling mechanisms linking the IGF and ERα pathways, combined targeted therapy has been suggested as a potential unique therapeutic strategy in breast cancer. For this reason, and following on from several preclinical studies, several trials targeting IGF1R in luminal breast cancer were performed. Unfortunately, anti-IGF1R therapy provided little benefit in this setting. It should be noted, however, that therapy was given to all patients independent of whether the IGF pathway was present or active (e.g., in the absence of biomarkers). Several *in vitro* studies suggest that biomarker selection is critical for identifying the tumors that will respond to IGF1R inhibitors (70, 83).

A major hurdle to anti-IGF1R therapy is the intricate network of feedback that occurs in this and related pathways. For example, the PI3K/Akt/mTOR/S6K pathway exerts a major negative feedback upon IGF1R/IRSs and when any part of this pathway is inhibited there is a concomitant increase in IGF1R activity. This was noted in Phase I trials of an mTOR inhibitor and validated in many preclinical studies (84). For example, inhibition of AKT in long-term estrogen deprived cell lines results in positive feedback that upregulates several upstream growth factor proteins through FoxO and ERα-regulated transcription, including IGF1R and IGF ligands (85). Combined inhibition of IGF1R/IR along with AKT inhibition and ER deprivation enhances the anti-tumor effect *in vivo* (85). The ability of the pathway to autoregulate and compensate for ER downregulation appears to be the cause for endocrine therapeutic resistance. The only way to impede resistance may be through inhibition of the overarching converging system, targeting multiple intercrossing growth signaling pathways to limit compensation on as many levels as possible.

### ERBB2-like

ERBB2 (HER2)-like tumors comprise ~15% of breast cancers and are typically hormone receptor negative with a 40% probability of p53 mutation (56). The ERBB2-like subtype shows poorer prognosis than luminal tumors, with early age of onset, higher tumor grade, and lymph node positivity. Patients with ERBB2-like tumors tend to have early recurrence and a poorer prognosis.

Accumulating evidence indicates crosstalk between ERBB2 and IGF signaling in breast tumorigenesis. About 10–20% of ERBB2^+^ tumors express IGF1R protein (62, 63). Active phosphorylated IGF1R/IR is found in 49% (86) and 64% (52) of ERBB2^+^ tumors. Brown et al. found phosphorylated IGF1R/IR does not correlate with prognosis in trastuzumab-treated ERBB2^+^ tumors (86). However, Yerushalmi et al. observed that ERBB2^+^ tumors expressing higher total IGF1R protein levels have decreased breast cancer specific survival compared to the lower IGF1R-expressing ERBB2^+^ counterparts (62). In this study, these ERBB2-enriched tumors are the only subtype presenting a low patient prognosis in correlation with IGF1R expression (62).

The risk of recurrence is higher for ERBB2 positive breast cancers than for ERBB2 negative breast cancers. This anti-ERBB2 therapy resistance is often due to activation of alternative growth factor receptor pathways. ERBB2-postive tumors expressing strong IGF1R membrane staining are less likely to respond to trastuzumab and vinorelbine than those with negative or low IGF1R protein expression (87). Other studies do not indicate a correlation between IGF1R protein expression and trastuzumab response (86, 88, 89) unless IGF1R expression is combined expression of downstream IGF1R signaling effectors, such as PI3K or mTOR (88).

Unlike clinical data, *in vitro* breast cancer cell line data suggest a strong correlation between increased IGF1R activity and trastuzumab-resistance (90–93). Interestingly, miRNAs, which typically inhibit IGF1R, show decreased expression upon trastuzumab-resistance (93, 94) and providing one possible mechanism of IGF1R upregulation. In trastuzumab-resistant cell line models, IGF1R forms a complex with ERBB2, and even a triplex with ERBB3 (95–97). This heterodimer/trimer promotes crosstalk between the growth receptor pathways. For example, IGF1-induced IGF1R phosphorylation leads to ligand-independent phosphorylation of ERBB2, which circumvents trastuzumab antibody inhibition and leads to an ERBB2-based mechanism of resistance (95, 98). In trastuzumab-resistant cells, IGF1R-promoted ERBB2 phosphorylation and IGF1R-induced invasion are mediated by Src and FoxM1 (98). Co-targeting ERBB2 and IGF1R reduces Erk/AKT activation, cell proliferation, *in vitro* invasion, and xenograft tumor growth to a greater extent than targeting either receptor individually (98, 99). Interestingly, treating trastuzumab-resistant cells with metformin re-sensitizes cells by disrupting the ERBB/IGF1R complexes (97), again strongly suggesting that a combined therapy would hold promise for patients with ERBB2^+^ breast tumors.

### Triple negative breast cancer

The triple negative breast cancer (TNBC) subtype accounts for up to 10–20% of breast cancers. Approximately 75% of TNBCs have a basal-like phenotype (56). Most BRCA-mutant tumors fall into this subtype (100). TNBCs are defined by the absence of ER, PR, and ERBB2. Driver mutations and subsequent targeted therapies are currently unknown. These tumors tend to be high grade and poorly differentiated with high rates of recurrence and poor prognosis. Intriguingly, TNBCs respond well to neoadjuvant chemotherapy with high rates of pathological complete response as compared to response in other subtypes. However, TNBCs still trend toward a poorer prognosis with increased rates of recurrence. The disproportion between response and outcome suggests neoadjuvant therapies are not capable of abolishing the driving tumorigenic cell types, underlining the need for identifiable and targetable driver mutations in TNBC.

Women of African descent are three times more likely to have TNBC: 30% of breast cancers diagnosed in African-American women are TN as compared to 11–13% of non-African-American women (101–103). Interestingly, African-American women have higher IGF1R expression in normal breast tissue while Caucasian-Americans have higher levels of IGF2R (104). This differential IGF1R/IGF2R expression may explain the increased occurrence of the more aggressive TNBC subtype in African-American women. Although IGF1R levels are similar between normal and malignant African-American breast tissues, phosphorylation of IGF1R and its downstream effectors are significantly higher in the malignant samples (104). Consequently, IGF1 signaling and proliferation (detected by gene expression profiling) are higher in TNBCs from African-American women compared to European-American (51). These studies underscore the significance of IGF1R in TNBC.

About 22–46% of TNBCs express IGF1R protein (52, 62, 63) and this expression correlates with shorter survival (105). The IGF1 gene signature correlates with expression signatures of TNBC tumors and cell lines (106) where both sample types are responsive to IGF1 signaling, promoting proliferation, and cell survival (107).

Laboratory studies analyzing anti-IGF1R therapeutic response typically demonstrate a favorable response to TNBC therapies. We demonstrated that TNBC cell lines and a primary tumor xenograft are sensitive to the anti-IGF-IR/InsR tyrosine kinase inhibitor BMS-754807 (106). Surprisingly, expression of a dominant-negative IGF1R during MMTV-Wnt1-mediated tumorigenesis accelerates mammary tumor formation and promotes aggressiveness (108). Interestingly, these tumors possess IGF2 signaling as well as a suggested role for InsR signaling. Additional studies demonstrate that IGF1R inhibition does not abrogate IGF-induced phenotypes in the presence of increased IGF2/IGF2R signaling (109). In TCGA patient data, IGF2R expression is significantly higher in basal-like tumors as compared to luminal tumors (Figure 2) (*p* value <0.001, *t*-test). Taken together, these studies suggest that IGF1R inhibition may be beneficial in some triple negative breast cancers but that the benefit will be very context-dependent.

Recently, the G protein estrogen receptor 1 (GPER/GPR30) has been identified as a potential growth regulator of TNBCs (110, 111). GPER is believed to mediate rapid estrogen response independently of ER; and thus, can drive estrogen-responsive growth even in ER-negative cells. As mentioned above, IGF1 signaling induces GPER expression and GPER promotes IGF1-induced migration and proliferation (77, 78). More work need to be completed in this area to determine if GPER could be a potential biomarker for anti-IGF1R-responsive TNBCs.

Most BRCA1 tumors phenocopy TNBC (100). In line with BRCA1-mediated repression of the IGF1R promoter (112, 113), BRCA1-mutant tumors show elevated IGFIR and IGF1 levels, leading to reduced apoptosis, and enhanced survival (113–115). Importantly, inhibition of the IGF1R/PI3K/AKT pathway decreases proliferation in BRCA1-deficient cells (116). These studies suggest IGF1R signaling significantly contributes to tumor cell proliferation and survival in BRCA1-deficient breast cancers.

## The Influence of IGF1R on Cell Potential and Cell Fate

### IGF1R signaling and stemness

The IGF system regulates stem cell maintenance in normal tissue processes. In human embryonic stem cells, the stem cell niche produces IGF2, which is required for survival and expansion (117). In neural stem cells, IGF2 is believed to bind and act through the InsR-A rather than IGF1R (118). Conversely, the human embryonic niche relies on the IGF2/IGF1R axis for self renewal and stem cell expansion (117), suggesting the necessity of IGF1R-promoted signaling in maintaining the stem cell population. In the hematopoietic and muscular system, expression of a skeleton muscle-localized IGF1 transgene enhances skeletal muscle regeneration in irradiated mice in part by recruiting proliferating bone marrow-derived cells, increasing stem cell marker populations, and accelerating myogenic differentiation (119, 120). Additionally in the hematopoietic system, IGF1R levels in newborn umbilical cord blood correlates positively to the total number of hematopoietic stem and progenitor cells (121). These studies demonstrate a role for IGF1R in regulation of stem and progenitor cell populations.

A recent review published by Roberta Malaguarnera and Antonio Belfiore summarized in great detail the known pathways and links between the IGF1R pathway and the epithelial-to-mesenchymal transition (EMT) and stem cell-related processes across several tissue types, both normal and cancerous (122). EMT is a naturally occurring process for remodeling of tissues and wound healing where polarized epithelial cells lose adherence and gain mesenchymal characteristics, including enhanced mobility and matrix invasion. Tumors often undergo EMT in an out-of-context manner. Interestingly, cells undergoing EMT also acquire stem cell-associated characteristics such as the capacity for self-renewal, gain of specific gene expression changes and cell surface markers, and ability to initiate tumorigenesis (123). The gain of these stem cell-associated properties suggests an overlap between EMT and stem cell mechanisms. In cancerous tissues, these overlapping mechanisms, now activated out-of-context, may play key roles in metastatic spread and resistance to cancer treatments.

Insulin-like growth factor 1 signaling is an integral part of both the EMT and stem cell-related processes in normal and cancerous tissues. Numerous *in vitro* studies demonstrate IGF1R as a driver of self renewal, stem cell surface markers, migration, and invasion in both normal and cancerous tissues and tumor initiation in hepatic, lung, prostate, and breast cancers (122). Each of these studies has begun to reveal the mechanisms of IGF1R-regulated EMT and stem phenotypes. Stem-promoting signaling pathways such as Wnt/B-catenin (124–127), Notch (128–130), and Shh (131, 132) act upstream to increase IGF1R expression with cross talk and regulation at the IGF1R promoter level by Sp1 and HMGA1 (133–136). In addition to upstream regulation by these master stem cell master controllers, IGF1R promotes positive downstream feedback through regulation and interaction with the well known EMT and stemness-linked transcription factors Zeb1 (137), NFκB (138), Snail (138), Twist (139), and p53, Sox2, Oct4, Nanog (140–142). Additionally, the tumor suppressor p53, known for inhibition of many stem cell regulators, inhibits IGF1R (143), which in turn act to downregulate p53. Interaction with these numerous stem-related pathways and factors strongly supports the central role of IGF1R in both the induction and the maintenance of stemness and EMT.

This role for IGF1R in promotion of stem-like characteristics has been specifically demonstrated in primary breast cancer. In human primary breast cancer xenografts, total and phosphorylated IGF1R expression is significantly higher in the CD44^+^CD24^−^ sorted breast cancer stem cell population compared to the non-CD44^+^CD24^−^ population (144). IGF1R expression and upregulated AKT activity are required for maintenance of this population. IGF1R inhibition reduces the aldehyde dehydrogenase^+^ stem-like population and suppresses mammosphere-forming capacity. Notably, silencing of IGF1R reduces tumor initiating ability of the xenografts. (144). Together this data demonstrate an active role for IGF1R in driving mammary stem-like phenotypes *in vitro* and *in vivo*.

### IGF1R and lineage fate

In addition to stem cell maintenance, two new studies suggest a link between IGF1R and cell lineage fate. In thyroid tissues, cell lineage differentiation is associated with IGF pathway activation (145). A similar trend is observed in neural cells. The lineage restricted neural progenitors primarily express IGF1R while the neural stems cells appear to rely more heavily on IGF2 and IR-A signaling (118). These studies suggest that IGF1R may be responsible for promoting cell fate, or at the very least, be restricted to maintaining differentiated cells of a specific lineage.

Morphogenesis and homeostasis of the mammary gland relies on stem cell function for the production and maintenance of the myoepithelial and luminal lineages. The TEBs contain the mammary gland stem cell niche and is where lineage differentiation and ductal morphogenesis occurs. IGF1 signaling promotes the development of the TEB. TEB formation and ductal outgrowth are grossly impaired in IGF1^−/−^ mice (26), signifying that IGF1 signaling is significant for development and/or maintenance of the mammary stem cell niche. While IGF1R-null mice die postnatally before mammary gland development, mammary gland transplantation of embryonic IGF1R-null mammary buds shows reduced ductal growth similar to IGF1-null mice (28). The IGF pathway is also important in pregnancy and lactation where luminal differentiation is vital. During early pregnancy, alveolar differentiation is reduced in heterozygous IGF1 mice (146). This same lack of alveolar budding and decreased alveolar density is observed in transgenic mice containing a pregnancy-induced kinase-dead IGF1R (147). Thus, the IGF pathway seems to have a role not only in mammary gland stem cell maintenance but also in lineage specification throughout the many stages of mammary gland development.

### IGF1R and breast cancer lineages

Breast tumors show both intra- and inter-tumor heterogeneity, suggesting distinct tumor initiation and progression pathways for each tumor type. Breast tumor heterogeneity likely results from a combination of both clonal outgrowth and aberrant differentiation of progeny. Defining the breast cancer cell of origin is a topic of major interest. Based on the cell of origin hypothesis, the heterogeneity of the tumor is restricted by a strictly linear differentiating breast cell hierarchy. Basically, the characteristics and differentiation potential of the tumor cells are restricted to the characteristics and differentiation potential of the cell of origin, which, in this case, is typically considered as a stem or progenitor cell. A variation on this cell of origin concept is that the tumor phenotype and potential is at least partially, if not primarily, determined by the genetic alterations acquired and not solely on the mammary lineage of origin. This variation suggests that the gained alterations activate or deactivate pathways out of context to alter a cell’s capabilities for differentiation and cell fate. Whether the heterogeneity is determined by the cell of origin or the genetic alterations or a mixture of explanations, the underlying importance is in understanding the potential and fate of the individual tumor cells.

Type 1 insulin-like growth factor receptor’s role in mammary stem cell maintenance and necessity for lineage differentiation suggests that aberrantly expressed IGF1R may be capable of enhancing cell potential and altering cell fate in a tumor, perhaps even in tumors composed of fully differentiated cells. As discussed above, IGF1R expression is essential for driving luminal alveolar differentiation, linking IGF1R to the luminal lineage. In breast cancer, IGF1R expression correlates most strongly with luminal breast cancers (60, 61). This expression may be a result of ER-driven growth through IGF1R rather than a causative link between IGF1R and the luminal lineage; nevertheless, the presence of IGF1R may still affect cell signaling and perhaps cell lineage. In addition to luminal tumors, IGF1R actively promotes tumor growth and survival in p53 and BRCA1-mutant tumors, which usually emulate the basal-like subtype (113–115). Although basal-like breast cancers are defined by basal and myoepithelial marker expression (148), they present with a luminal progenitor gene signature (148–152). In support of this luminal link, recent studies suggest BRCA1-associated basal-like tumors derive from a luminal progenitor cell of origin rather than a basally positioned cell (150, 153). Elevated IGF1R expression and signaling in these basal-like tumors appear to have active roles in tumor promotion. Thus, as IGF1R is associated with cell lineage fate, IGF1R signaling may be influencing the gain and loss of lineage markers and phenotypes in these tumors. Taken all together, these data suggest that IGF1R may be connected with driving lineage fate, particularly luminal-associated fate.

## Therapeutic Targeting of IGF1R as an Anti-Cancer Therapy

Based upon extensive basic, preclinical, and clinical evidence, a range of anti-IGF1R therapeutic strategies have been developed, including humanized monoclonal antibodies, which prevent ligand binding and small-molecule inhibitors that inhibit the tyrosine kinase domain. Early clinical data from anti-IGF1R trials were very promising. In a Phase I trial of the monoclonal antibody, AMG 479, a patient with chemo-refractory Ewing sarcoma had complete remission (154). In a Phase II trial, 14% of 125 patients with recurrent or refractory sarcoma responded to the monoclonal IGF1R antibody, R1507 (155, 156). In both of these trials, therapy was well tolerated. In contrast, two large NSCLC Phase III trials of figitumumab in combination with either carboplatin and paclitaxel or Tarceva, respectively, were terminated due to lack of response and severe toxicities (157, 158).

The lack of response in anti-IGF1R trials has been a large concern. The failure may be a consequence of trial design and not the efficacy of the IGF1R inhibitors themselves (159). In the Phase III trials, patients were not screened for IGF1R expression, limiting the percent of patients that even had a chance to respond to the therapy. Additionally, monoclonal antibody inhibition is specific to IGF1R and does not inhibit the InsR, which can also stimulate tumorigenesis. InsR signaling increases upon IGF1R inhibition, suggesting pathway compensation (160–163). As touched on throughout the above review, there is also substantial crosstalk between many of the growth factor signaling pathways, including IGFR, EGFR, ErbB2, and ERα. A combinatory therapy approach may be needed for efficient suppression without compensation or resistance.

Before continuing anti-IGF1R therapies, it is necessary to define additional IGF1R-based biomarkers to more accurately predict anti-tumor response and identify responsive tumors. A better understanding of the important components of the IGF1R signaling pathway and the instances where crosstalk and compensation can occur is required. Only then can we pinpoint the cancer subtypes that will benefit from IGF1R therapeutics, alone or in combination with other inhibitors.

## Overview

Type 1 insulin-like growth factor receptor plays a key role in cancer promotion, resistance, and recurrence across breast cancer subtypes. Understanding the role of IGF1R in individual subtypes is critical to better targeting. The current research highlights IGF1R as necessary for stem cell maintenance and expansion. In turn, additional studies demonstrate the need and expression of IGF1R in more differentiated populations. Taken together, these studies suggest that IGF1R may not only be driving stem cell characteristics, leading to increased therapy resistance, but also that altered IGF1R signaling in tumor cells may influence expression of lineage-linked traits and direct lineage potential and fate, contributing to the heterogeneity of tumors.

## Conflict of Interest Statement

The authors declare that the research was conducted in the absence of any commercial or financial relationships that could be construed as a potential conflict of interest.
